# The relationship between the Charlson Comorbidity Index and anorexia in older adults: the mediating role of depressive symptoms

**DOI:** 10.3389/fnut.2026.1753095

**Published:** 2026-03-30

**Authors:** Haichen Wu, Pengxin Dong, Yidan Chai, Ping Huang, Lichong Lai, Jie Peng, Xiaoying Cao, Xiaoling Feng, Zhixin Li, Haowei Liu, Jingyun Zeng, Huimin Zhou, Dongmei Huang, Huiqiao Huang

**Affiliations:** The Second Affiliated Hospital of Guangxi Medical University, Nanning, China

**Keywords:** anorexia of aging, Charlson Comorbidity Index, depressive symptoms, older adults, PHQ

## Abstract

**Objective:**

To examine the association between the Charlson Comorbidity Index and anorexia of aging and to test whether depressive symptoms mediate this relationship.

**Methods:**

In November 2024, 382 older adults were recruited from three economic regions of Guangxi using multi-stage stratified sampling. Logistic regression identified risk factors, and a mediation model was constructed to test whether depressive symptoms mediate the relationship between the Charlson Comorbidity Index and anorexia of aging.

**Results:**

Anorexia prevalence was 26.7%. Charlson Comorbidity Index was a risk factor for anorexia of aging (OR = 2.835, 95% CI 1.693–4.746), and the PHQ-9 score was also associated with anorexia (OR = 1.179, 95% CI = 1.077–1.291). The indirect effect via PHQ-9 accounted for 17.8% of the total effect; when depression was dichotomised, the mediated proportion was 12.3%.

**Conclusion:**

Comorbidity burden increases the risk of anorexia of aging directly and indirectly by inducing depressive symptoms. Simultaneous management of chronic diseases and negative affect is warranted to prevent anorexia of aging and its downstream adverse health consequences.

## 1 Introduction

With the global population aging accelerating, preventable health conditions common to older adults demand urgent attention. One such geriatric syndrome is anorexia of aging (AA), characterized by a clinically relevant loss of appetite and reduced food intake, not attributable to acute illness, but intrinsic to the aging process itself (1). AA is multidimensional in origin, driven by concurrent changes in physiological energy-balance pathways, reward-system neuropsychology, and sociocultural cues for eating (2). Community studies report a prevalence of 3.4–33% (3–6), yet the condition is frequently overlooked or dismissed as “normal” aging, leading to delayed diagnosis and intervention (7). Beyond impairing quality of life, AA is significantly associated with weight loss (8), malnutrition (9), sarcopenia (10, 11), frailty (3) and increased mortality (9, 12), thereby imposing substantial health and economic burdens on individuals and healthcare systems (13).

The pathogenesis of anorexia of aging involves a complex interplay of physiological, psychological, and social factors. These include a decline in appetite-regulating hormones, increased levels of glucagon-like peptides and cholecystokinin, and reduced gastrointestinal motility (14). Other contributing factors are chronic low-grade inflammation (15), gut microbiota dysbiosis (13), impaired sensory functions such as taste and smell (16), and chewing difficulties (8). Additionally, older age (10), female sex (8), depressive symptoms (17), living alone (18), low cognitive function (10), smoking (10), physical inactivity (10), and low socioeconomic status (10, 19) have also been associated with the development of anorexia in older adults.

Comorbidity refers to the presence of two or more medical conditions in the same patient, either concurrently or sequentially within a given time frame (20). It is commonly assessed using the Charlson Comorbidity Index (CCI). Among patients with chronic diseases, higher CCI scores are strongly associated with the development of depressive symptoms (21) and are also significantly linked to anorexia of aging (18). For instance, gastrointestinal disorders, malabsorption syndromes, acute and chronic infections, hypermetabolic states, as well as malignancies and rheumatoid arthritis can all contribute to the onset of anorexia (22). Anorexia correlates with the Charlson Comorbidity Index (22, 23). Comorbidities not only exacerbate the severity of anorexia in older adults but may also increase mortality risk by compromising overall health status and physical function, leading to a cascade of adverse outcomes such as malnutrition and immune dysfunction. However, the exact relationship between CCI and anorexia of aging remains unclear. Individuals with anorexia are more likely to develop depression than those without (17), raising the question of whether depression mediates the association between CCI and anorexia.

Therefore, investigating the mediating role of depression in the relationship between comorbidity burden and anorexia may provide a scientific basis for the early identification of high-risk individuals and the development of targeted interventions, ultimately preventing the chain of adverse health outcomes triggered by anorexia in aging and promoting healthy aging.

## 2 Methods

### 2.1 Participants

A multi-stage stratified sampling strategy was employed in November 2024.

Stage 1 – Administrative stratification: All cities within Guangxi Zhuang Autonomous Region were ranked by per-capita GDP and classified into high, medium, or low economic tertiles. One city (primary sampling unit, PSU) was randomly selected from each tertile. Within each selected city, one county and one urban district (secondary sampling units, SSU) were chosen by simple random sampling.

Stage 2 – Community selection: One township within the sampled county and one urban community within the sampled district were randomly selected as tertiary sampling units (TSU).

Stage 3 –Participant recruitment: To recruit older adults, we collaborated with local community health service centers in each selected township and urban community. Complete lists of permanent residents aged 60 years and above were obtained from their administrative records, and potential participants were selected using simple random sampling. Invitation methods included telephone contact; for residents without telephone access or who could not be reached by phone, trained community health workers conducted home visits to extend invitations. Data collection was conducted at the local community health service centers. Prior to participation, each participant was provided with detailed information regarding the study purpose, procedures, potential risks and benefits, confidentiality measures, and the right to withdraw at any time.

Inclusion: (1) age ≥ 60 years; (2) registered local resident or having lived in Guangxi for ≥ 6 months; (3) able to communicate verbally and willing to provide written informed consent.

Exclusion: (1) critical illness (e.g., decompensated liver/renal failure, respiratory failure, advanced malignancy) that precluded interview; (2) withdrawal before completion of all assessments.

The prevalence of anorexia of aging among community-dwelling older adults ranges from 3.4 to 33%. Based on the sample size formula: *n* = *Z_α_*^2^ × *P*(1-*P*)/*δ*^2^, *α* = 0.05, *Z* = 1.96, *δ* = 0.05, the calculated sample size was 340. After accounting for a 10% non-response or invalid questionnaire rate, the target sample size was 378. A total of 382 valid questionnaires were obtained. All participants provided informed consent. The study was approved by the Research Ethics Committee of the Second Affiliated Hospital of Guangxi Medical University (approval No. 2023-KY-0905).

### 2.2 Measurement

#### 2.2.1 CCI measurement

The Charlson Comorbidity Index (CCI) is a validated instrument for quantifying the burden of comorbid disease in clinical and epidemiological research (24). This index assesses the health burden of patients by considering the number and severity of various chronic diseases. With the development of medicine, the Charlson Index has been revised and now includes a variety of chronic diseases, such as chronic lung disease, rheumatism, diabetes with chronic complications, kidney disease, congestive heart failure and liver disease (25). In this study, trained interviewers administered a standard medical-history questionnaire. CCI covers chronic conditions relevant to older adults, and has been widely used in geriatric research examining the relationship between physical health and psychological outcomes, making it particularly suitable for community-based surveys in this population. Participants responded to the item “Has a doctor ever diagnosed you with any of the following conditions?” Unchecked or missing items were coded as “absent” and assigned a weight of 0. Total CCI scores were computed for each participant and then dichotomised for analysis: CCI = 0 (no comorbidity) and CCI > 0 (at least one comorbidity).

#### 2.2.2 Depression measurement

The 9-item Patient Health Questionnaire (PHQ-9) is a validated screening instrument for depressive symptoms. Each item corresponds to one DSM-5 criterion and is scored 0–3 according to the frequency of the symptom during the previous 2 weeks: 0 = “not at all,” 1 = “several days,” 2 = “more than half the days,” 3 = “nearly every day.” Total scores range from 0 to 27; higher scores indicate greater symptom severity. Consistent with standard cut-offs, participants with a total score ≥ 5 were classified as having probable depression (26). The PHQ-9 has demonstrated good reliability and validity in both older adult populations and Chinese populations. Its 9-item format maintains strong psychometric properties while minimizing respondent burden, making it particularly suitable for community-based surveys with older adults.

#### 2.2.3 Anorexia of aging measurement

The Simplified Nutritional Appetite Questionnaire (SNAQ) is a validated 4-item tool designed to screen for anorexia in older adults (27). Each item is scored 1–5, yielding a total range of 4–20; higher scores denote better appetite. Following the established cut-off, participants with a total score ≤ 14 were classified as having anorexia and considered at high risk of malnutrition (27). The SNAQ has a brief format suitable for community-based surveys in older adults.

### 2.3 Covariates

This study collected comprehensive baseline characteristics: age, sex, residence, education, marital status, monthly income, smoking and alcohol history, weekly physical-exercise frequency, anthropometric measures (height, weight, waist, mid-upper arm and calf circumferences, grip strength), and cognitive function.

### 2.4 Statistical analysis

Data were analyzed with SPSS 25.0 and R 4.3.0. Continuous variables were expressed as mean ± SD or median (inter-quartile range) according to distribution, and categorical variables as *n* (%). Normality was examined with the Shapiro–Wilk test. Group comparisons were performed with the *χ*^2^ or Fisher’s exact test for categorical variables, one-way ANOVA for normally distributed continuous variables, and the Kruskal–Wallis test for skewed variables. Logistic regression was applied to identify factors associated with anorexia after adjusting for age, Frequency of physical exercise per week, Arm circumference, hip circumference, Calf circumference, BMI (kg/m^2^), Grip strength, and cognitive function. The mediation package in *R* was used to test whether depressive symptoms mediated the association between CCI and anorexia. Bias-corrected 95% confidence intervals for the indirect effect were estimated using 5,000 bootstrap resamples; significance was assumed if the CI did not include zero. A two-sided *p*-value of less than 0.05 was considered statistically significant.

## 3 Results

### 3.1 Basic characteristics of the participants

A total of 382 participants were included and classified by anorexia status (Table 1). Anorexia was present in 102 individuals (26.7%). Compared with those without anorexia, participants with primary-school education or below accounted for 56.9% of the anorexia group, and the proportion was similarly high in those without a fixed income. Exercise frequency was significantly associated with appetite loss: 43.1% of participants exercising ≥ 3 times per week had anorexia. Anthropometric measurements were lower in the anorexia group for upper-arm circumference, hip circumference, BMI, calf circumference and grip strength. Depressive symptoms were present in 32.4% of participants with anorexia. Cognitive impairment was also more frequent in this group (*p* < 0.05).

**Table 1:** Basic characteristics of participants and one-factor analysis.

Variables	Total [*n* = 382 (%)]	Anorexia [*n* (%)]		*x^2^/Z*	*p*
		Yes	No		
Age (years)				7.550	0.023
60–69 years	156 (40.8%)	30 (29.4%)	126 (45.0%)		
70–79 years	143 (37.4%)	45 (44.1%)	98 (35.0%)		
≥80 years	83 (21.7%)	27 (26.5%)	56 (20.0%)		
Residence				0.403	0.525
City	177 (46.3%)	50 (49.0%)	127 (45.4%)		
Rural	205 (53.7%)	52 (51.0%)	153 (54.6%)		
Gender				0.002	0.969
Male	123 (32.2%)	33 (32.4%)	90 (32.1%)		
Female	259 (67.8%)	69 (67.6%)	190 (67.9%)		
Marital status				0.811	0.368
Married	267 (69.9%)	70 (68.6%)	197 (70.4%)		
No married	115 (30.1%)	32 (31.4%)	83 (29.6%)		
Living arrangements				0.025	0.874
Cohabitation	335 (87.7%)	89 (87.3%)	246 (87.9%)		
Living alone	47 (12.3%)	13 (12.7%)	34 (12.1%)		
Educational period				6.18	0.103
Primary education or below	204 (53.4%)	58 (56.9%)	146 (52.1%)		
Junior high school	96 (25.1%)	30 (29.4%)	66 (23.6%)		
High school/Secondary vocational school	64 (16.8%)	12 (11.8%)	52 (18.6%)		
College degree or above	18 (4.7%)	2 (2.0%)	16 (5.7%)		
Per-capita monthly income				6.033	0.110
No fixed income	140 (36.6%)	46 (45.1%)	94 (33.6%)		
<1,000 CNY	107 (28.0%)	24 (23.5%)	83 (29.6%)		
1,000–3,000 CNY	66 (17.3%)	19 (18.6%)	47 (16.8%)		
>3,001 CNY	69 (18.1%)	13 (12.7%)	56 (20.0%)		
Frequency of physical exercise per week				32.841	<0.001
0 times/week	79 (20.7%)	31 (30.4%)	48 (17.1%)		
1–2 times/week	53 (13.9%)	27 (26.5%)	26 (9.3%)		
≥3 times/week	250 (65.4%)	44 (43.1%)	206 (73.6%)		
Smoking status				0.171	0.918
Current smokers	40 (10.5%)	10 (9.8%)	30 (10.7%)		
Non-smokers	329 (86.1%)	89 (87.3%)	240 (85.7%)		
Former smokers	13 (3.4%)	3 (2.9%)	10 (3.6%)		
Drinking status				4.519	0.104
Current drinkers	38 (9.9%)	5 (4.9%)	33 (11.8%)		
Non-drinkers	334 (87.4%)	94 (92.2%)	240 (85.7%)		
Former drinkers	10 (2.6%)	3 (2.9%)	7 (2.5%)		
Anthropometry					
Arm circumference (cm)	26.0 (24.0, 28.0)	25.0 (23.0, 28.0)	26.0(24.0, 29.0)	−2.818	0.005
Waist circumference (cm)	84.0 (78.0, 90.0)	83.0 (78.0, 88.0)	85.0 (78.0, 90.0)	−1.421	0.155
Hip circumference (cm)	93.0 (88.0, 98.0)	92.0 (86.0, 96.3)	93.5 (88.0, 98.0)	−1.978	0.048
Calf circumference (cm)	31.0 (28.0, 34.0)	30.0 (28.0, 33.0)	31.8 (29.0, 34.8)	−3.069	0.002
BMI (kg/m^2^)	22.7 (20.0, 24.8)	22.1 (19.3, 24.4)	22.9 (20.4, 25.0)	−2.029	0.042
Grip strength (kg)	20.2 (14.8, 25.9)	18.2 (12.4, 22.6)	21.0 (15.7, 26.7)	−3.303	<0.001
Charlson Comorbidity Index				15.028	<0.001
=0	254 (66.5%)	52 (51.0%)	202 (72.1%)		
>0	128 (33.5%)	50 (49.0%)	78 (27.9%)		
Cognitive function				9.821	0.002
No	247 (64.7%)	53 (52.0%)	194 (69.3%)		
Yes	135 (35.3%)	49 (48.0%)	86 (30.7%)		
Depressive symptom·				29.124	<0.001
No	322 (84.3%)	69 (67.6%)	253 (90.4%)		
Yes	60 (15.7%)	33 (32.4%)	27 (9.6%)		
PHQ-9	1.0 (0.0, 3.0)	3.0 (1.0, 5.0)	1.0 (0.0, 3.0)	−5.699	<0.001

Data are presented as mean ± standard deviation, or percentage (%).

BMI: ratio of weight to height squared, PHQ-9: Patient Health Questionnaire-9, *p*-values < 0.05 are considered statistically significant.

In the fully adjusted model, both the Charlson Comorbidity Index (OR = 2.835 [1.693, 4.746]) and the PHQ-9 score (OR = 1.179 [1.077, 1.291]) were independent risk factors for anorexia (Tables 2, 3).

**Table 2:** Regression analysis of the Charlson Comorbidity Index and anorexia.

Variable	Non-adjusted	Adjust I	Adjust II
Charlson Comorbidity Index			
=0	ref	ref	ref
>0	2.490 (1.560, 3.976)***	2.750 (1.663, 4.548)***	2.835 (1.693, 4.746)***

Adjust I: Adjusted for Age, Frequency of physical exercise per week, Adjust II: Adjusted for Age, Frequency of physical exercise per week, Arm circumference, Hip circumference, Calf circumference, BMI (kg/m^2^), Grip strength, Cognitive function. ***p < 0.001.

**Table 3:** Regression analysis of depression, Patient Health Questionnaire-9 scores and anorexia.

Variable	Non-adjusted	Adjust I	Adjust II
Depression			
No	ref	ref	ref
Yes	4.481 (2.524, 7.957)***	3.504 (1.910, 6.428)***	2.998 (1.588, 5.659)***
PHQ-9	1.246 (1.147, 1.355)***	1.204 (1.105, 1.312)***	1.179 (1.077, 1.291)***

Adjust I: Adjusted for Age, Frequency of physical exercise per week, Adjust II: Adjusted for Age, Frequency of physical exercise per week, Arm circumference, Hip circumference, Calf circumference, BMI (kg/m^2^), Grip strength, Cognitive function. ***p < 0.001.

### 3.2 Analysis of mediation effects

**Figure 1:**
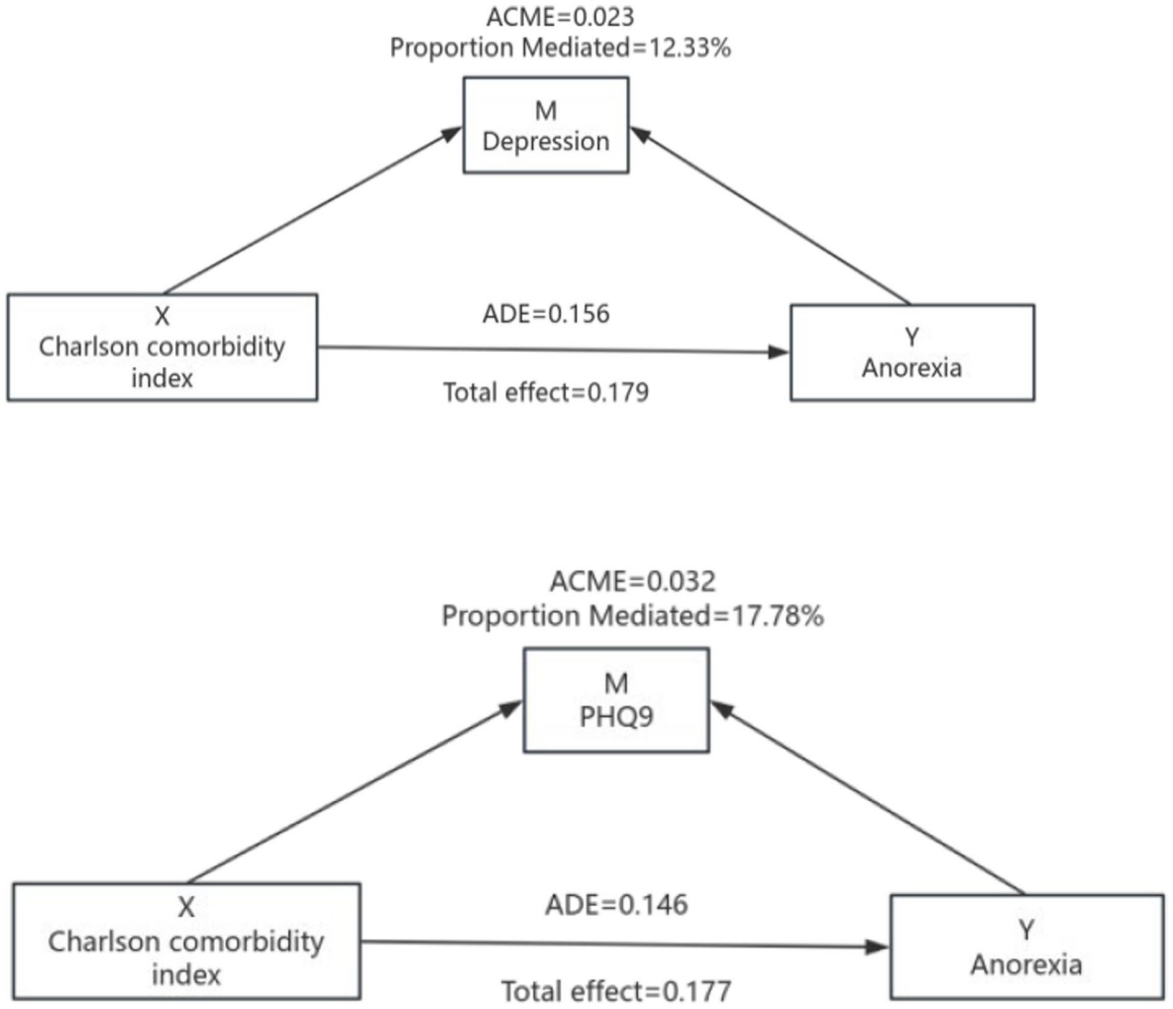
Mediation effect pattern diagram for assessing the mediation effect of depression after adjustment.

Based on the previous analysis, we examined whether depressive symptoms mediate the association between the CCI and anorexia of aging. After adjustment for confounders, mediation analysis showed a significant indirect effect via PHQ-9 score, with a coefficient of 0.032 (95% CI 0.009–0.059), accounting for 17.78% of the total effect. In the depression-mediated model, the total effect of CCI on anorexia of aging was 0.179 (95% CI 0.091–0.264), and the direct effect was 0.156 (95% CI 0.069–0.238). Thus, 12.33% of the total effect was mediated by depression, and the indirect effect remained statistically significant (Table 4 and Figure 1).

**Table 4:** Mediation analysis of PHQ-9 score and depressive symptoms in older adults.

Independent	Mediator	Total effect	Direct effect	Mediation effect	Mediation proportions
Charlson Comorbidity Index	PHQ-9	0.177 (0.091, 0.266)***	0.146 (0.059, 0.230)***	0.032 (0.009, 0.059)**	17.78
	Depression	0.179 (0.091, 0.264)***	0.156 (0.069, 0.238)***	0.023 (0.004, 0.053)*	12.33

PHQ-9: Patient Health Questionnaire-9. Adjusted for Age, Frequency of physical exercise per week, Arm circumference, Hip circumference, Calf circumference, BMI (kg/m2), Grip strength, Cognitive function. *p < 0.05, **p < 0.01, ***p < 0.001.

## 4 Discussion

This study aimed to examine the associations among the Charlson Comorbidity Index, depressive symptoms, and anorexia in older adults, and to investigate whether depressive symptoms mediate the relationship between Charlson Comorbidity Index and anorexia. Higher Charlson Comorbidity Index was a risk factor for anorexia, and depressive symptoms mediated 12.33% of the total effect.

This study found that the Charlson Comorbidity Index (CCI) is an important risk factor for anorexia in the elderly. The CCI covers a variety of disease states, among which cancer (28), chronic kidney disease (29), chronic heart failure (30) are common causes of anorexia in older adults. These diseases can reduce appetite by inducing mechanisms such as gastroparesis (31). In addition, chronic diseases are often accompanied by persistent systemic inflammatory responses; studies have shown that elevated levels of inflammatory markers such as interleukin-6 (IL-6), tumor necrosis factor-α (TNF-α) and C-reactive protein (CRP) can interfere with central appetite regulation (32). Chronic inflammation can also suppress appetite pathways by altering tryptophan metabolism and serotonergic activity, further leading to appetite loss (33). The polypharmacy required to manage multiple chronic diseases may cause adverse reactions such as nausea, dry mouth, and constipation, further exacerbating anorexia symptoms (34, 35). In summary, the combined effects of the diseases themselves and their treatment-related factors increase the risk of anorexia in the elderly.

This study examined the mediating role of depressive symptoms in the relationship between the Charlson Comorbidity Index and anorexia of aging. The results showed that depression acted as a partial mediator, suggesting that CCI may contribute to depressive symptoms, which in turn reduce appetite. The link between depression and appetite loss may be explained by several synergistic physiological mechanisms. Literature indicates that depression is associated with dysregulation of the neuroendocrine and autonomic nervous systems, as well as alterations in brain reward circuits, all of which can suppress appetite (31, 36). Depression is also linked to immune activation and elevated levels of pro-inflammatory cytokines (37). These inflammatory factors can overactivate indoleamine 2, 3-dioxygenase (a key enzyme in the kynurenine pathway), leading to decreased serotonin levels in the tryptophan-serotonin (5-HT) pathway. Serotonin is an important neurotransmitter involved in mood regulation; its reduction can exacerbate depression, and contribute to anorexia (38, 39). At the behavioral level, depressed individuals often exhibit behavioral characteristics such as weakened interest in food and decreased motivation to eat. These changes may also jointly contribute to the occurrence of anorexia. In conclusion, this study provides a new perspective for understanding anorexia of aging.

Psychological factors also contribute to anorexia in older adults. The eating disorder research has identified the phenomenon of the “eating disorder voice”—an internal dialog perceived by individuals as both protective and comforting, yet simultaneously controlling and intrusive. This voice often becomes a dominant psychological force, even perceived as more powerful than the individual’s own willpower, creating a constant struggle in which the person depends on it for emotional support while simultaneously desiring to escape its oppression (40). Patients commonly experience this voice as an external controller that reinforces restrictive eating through critical comments while offering a false sense of safety that prevents recovery. Closely related to this internal voice is the deficit in emotion regulation. Xiao et al. (41) found that emotion regulation difficulties significantly predicted eating disorder symptoms 1 year later in Chinese older adults, with “difficulty controlling impulses in negative emotional states” particularly associated with restrictive eating. For older adults with chronic diseases, illness-induced distress may trigger negative emotions; lacking effective emotion regulation strategies, individuals may use restrictive eating to obtain temporary emotional relief (42)— a mechanism that may mutually reinforce the internal critical voice: when the voice criticizes the self, negative emotions are activated; if these emotions cannot be regulated, individuals are more likely to submit to the voice’s commands. For older adults with multiple chronic conditions, the eating disorder voice may provide a sense of control amid chronic disease uncertainty (43); it may also reinforce restrictive eating patterns and exacerbate nutritional deficits.

Eating is inherently a social activity in Chinese culture, where sharing a meal is not only a process of nutritional intake but also a vehicle for emotional connection (44). Studies have shown that loneliness is associated with decreased appetite in older adults (45, 46). Social isolation and loneliness have been recognized as risk factors for the physical and mental health of older adults. Loneliness and social isolation are not merely consequences of poor health but can also influence physiological processes and health behaviors, including eating behavior (47). For older adults with chronic diseases, reduced mobility and shrinking social networks may disrupt established meal-sharing habits, transforming eating from a social activity into a solitary routine, thereby diminishing the sensory pleasure and motivation to eat. Loneliness may weaken one’s sense of purpose in life (48), reducing the willingness to prepare meals and maintain regular eating patterns. This state of “solitary eating” may further activate the inner critical voice. Furthermore, stigma is a significant psychological barrier that prevents older adults from seeking help. Older adults commonly perceive eating disorders as a “young person’s problem,” feel ashamed of their own eating difficulties, and lack confidence in whether medical professionals can understand their issues. This may lead to the long-term neglect of anorexia symptoms (49), delaying timely intervention.

This study has several limitations. First, the cross-sectional design precludes establishing causal relationships among the Charlson Comorbidity Index, depression, and anorexia; prospective cohort studies or randomized controlled trials are needed to clarify these pathways. Second, the sample size was relatively small and restricted to Guangxi, which may limit the generalizability of the findings; future studies should enroll larger, multi-region populations. Third, depression and anorexia were assessed by self-report questionnaires, introducing potential subjective bias; integrating multiple instruments and objective measures could enhance measurement accuracy.

## 5 Conclusion

The Charlson Comorbidity Index is significantly associated with anorexia in older adults, with depressive symptoms playing a partial mediating role. The findings suggest that physical comorbidity and mental health interact in the elderly, with depression potentially playing a key role in the onset and progression of anorexia. Therefore, focusing on both physical and mental health may help improve understanding and management of anorexia in older adults.

## Data Availability

The raw data supporting the conclusions of this article will be made available by the authors, without undue reservation.
